# Supplementary material for: A novel RofA-family transcriptional regulator, GadR, controls the development of acid resistance in Listeria monocytogenes
Source: mBio. 2023 Oct 26;14(6):e01716-23. doi: 10.1128/mbio.01716-23 (PMC10746197; doi:10.1128/mbio.01716-23)
Supplement: Supplemental data — Figures S1-S5; Tables S1 and S2. [file mbio.01716-23-s0001.docx]

**Supplemental Material**

**SFigures**

**
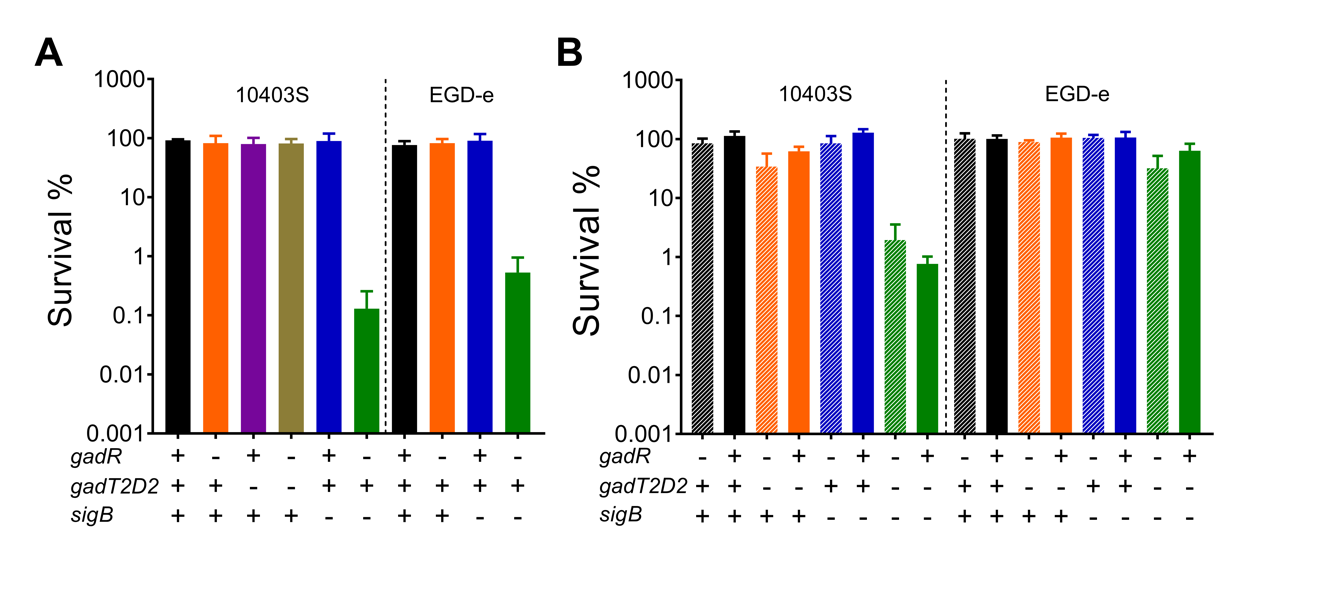
Fig S1. Treatment at pH 3.00 or pH 3.25 for 1 hour is not lethal to strains lacking either *gadR* or *sigB*.**

The strains examined in GABAe assay were grown to stationary phase and challenged at pH 3.00 (A) for 1 h or grown to exponential phase and challenged at pH 3.25 (B) for 1 h. Percentage survival values are presented. Each data point represents the average of three independent experiments performed with technical triplicates.

**
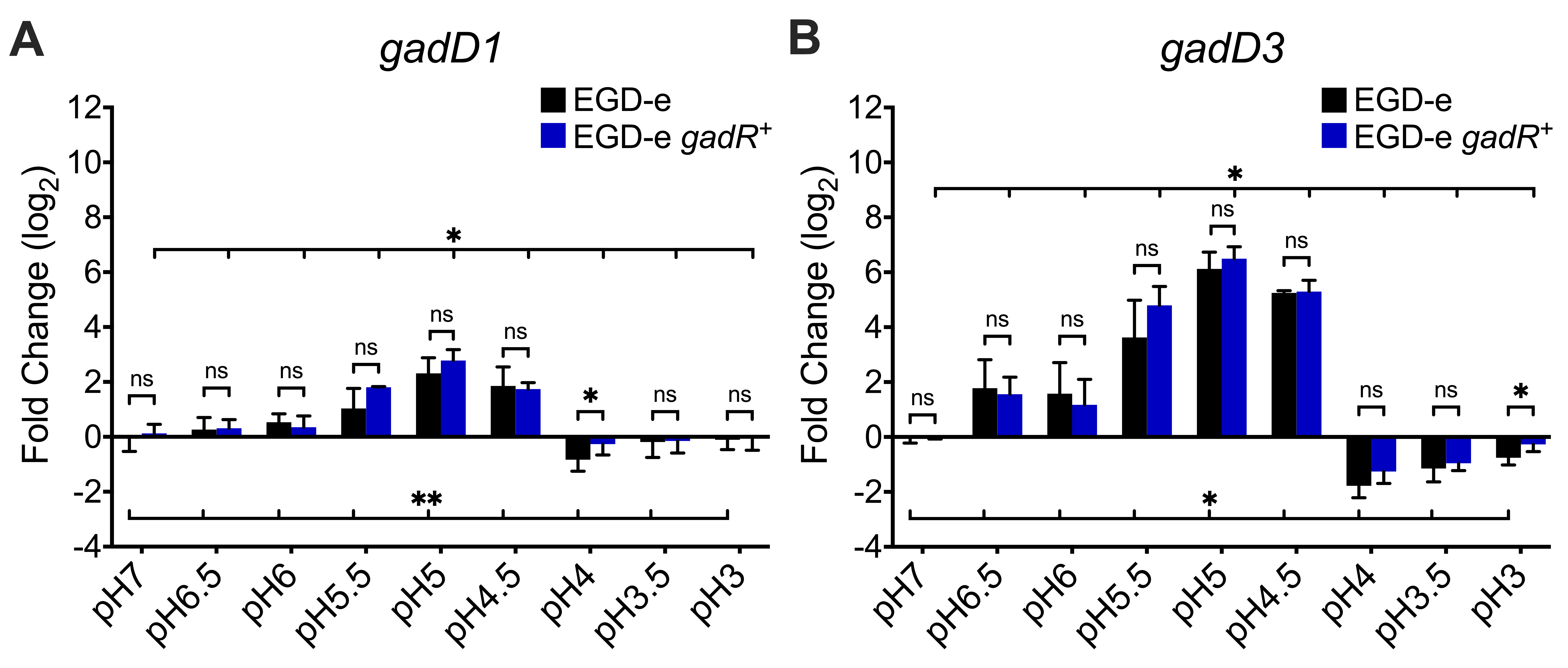
Fig S2. *gadD1* and *gadD3* are both induced by acid stress independently of GadR.**

The transcription levels of *gadD1* (A) and *gadD3* (B) in exponential phase cultures of EGD-e WT (*gadR*^-^) or *gadR*^+^ strains with or without a 15 min exposure to pH 3.0 - 6.5 are shown, expressed relative to untreated EGD-e WT stain. Three independent experiments were performed with technical duplicates. Statistically significant differences between two samples were determined using paired *t* test (two-tailed) (ns, not significant; * *p* < 0.05; **, *p* < 0.01).

**
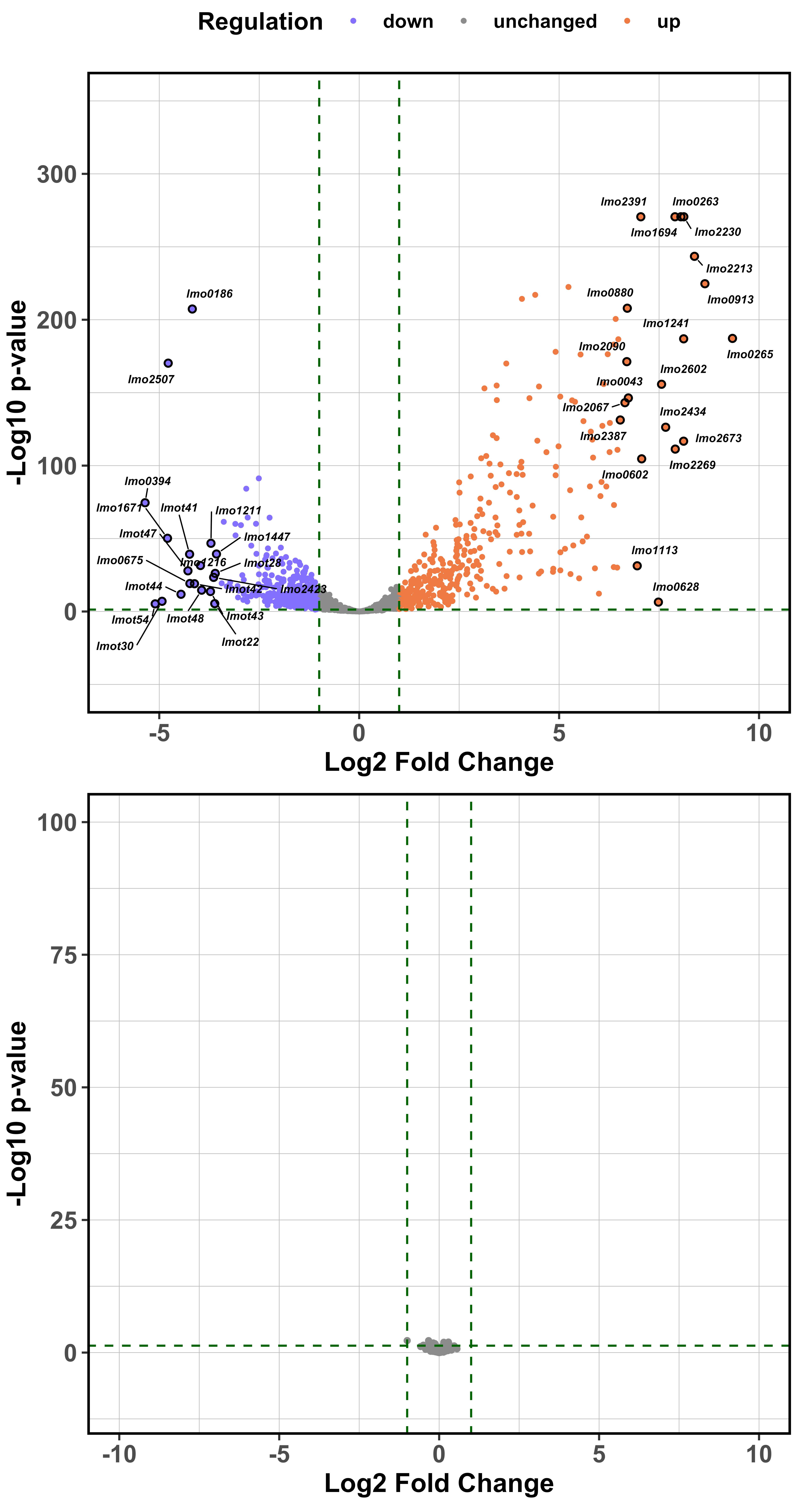
Fig S3. Acid stress results in global transcriptomic response in strain EGD-e while GadR does not play a significant role in gene transcription under exponential phase.**

**B**

**A**

(A) Global gene transcription following a 15 min pH 5.0 treatment was measured using RNA-Seq, with values expressed relative to the untreated exponential phase culture of strain EGD-e WT (*gadR*^-^). The 20 most upregulated/downregulated genes are labeled. (B) Global gene transcription of strain EGD-e *gadR*^+^ was compared to the WT EGD-e strain (*gadR*^-^) without pH 5.0 adaption. Genes showing differential transcription are labeled. Genes with differential transcription > 2 fold and with *p*-value < 0.05 were considered significantly differentially regulated (marked by dotted lines on the Volcano plots).

**
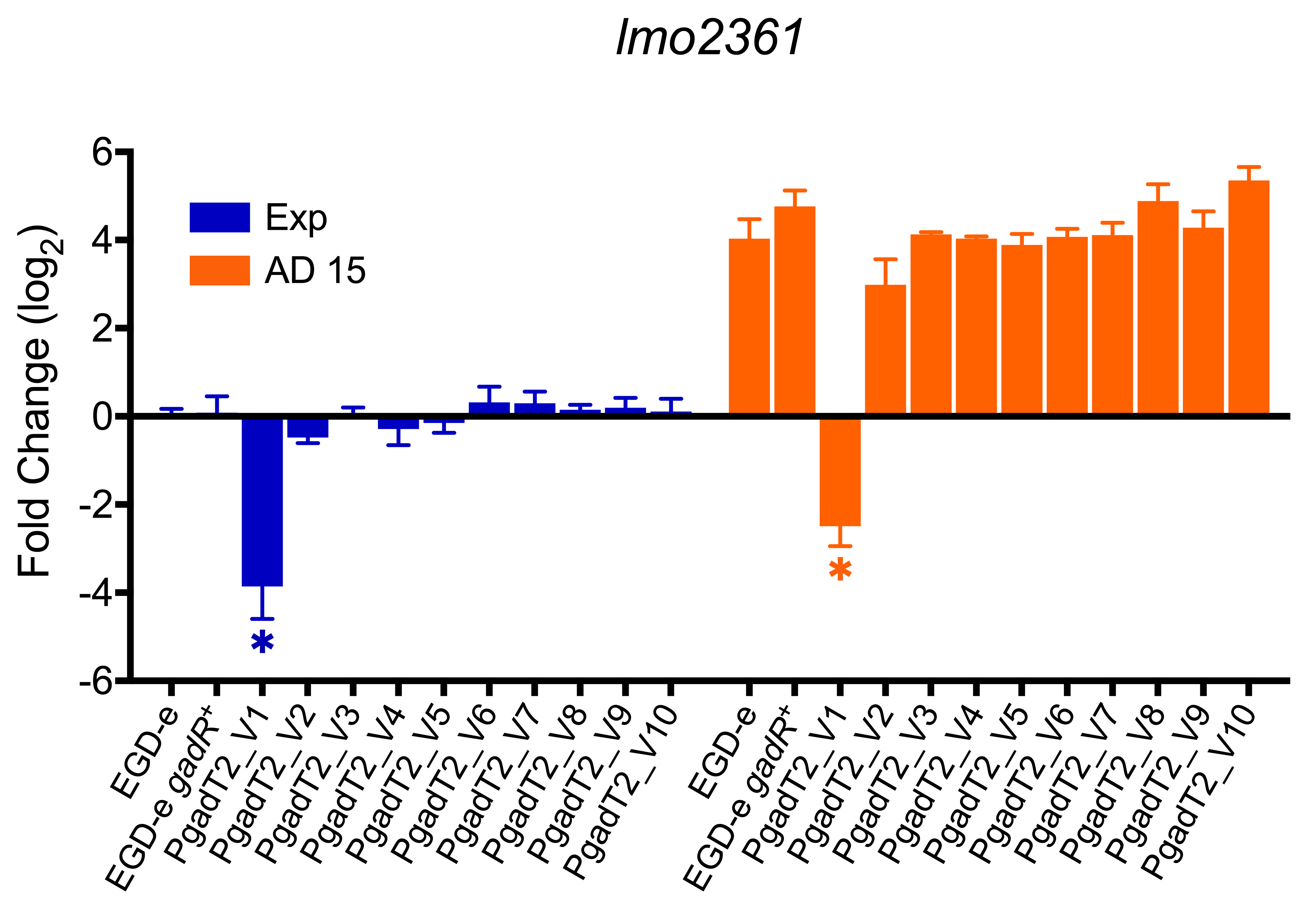
**

**Fig S4. *lmo2361*::*gadT2* intergenic sequence to 5’- end of putative GadR-boxes are required for *lmo2361* transcription.**

The transcription of *gadT2* was measured for the panel of P*gadT2* mutants during exponential phase either with (AD 15) or without (Non-AD) a 15 min pH 5.0 treatment and expressed relative to the untreated WT EGD-e strain (*gadR^-^*). EGD-e wild type strain at exponential phase without acid treatment was used a reference condition. Three independent experiments were performed with technical duplicates. Statistically significant differences across samples without acid treatment or with acid treatment were determined by one-way ANOVA (ns, not significant; * *p* < 0.05).

**
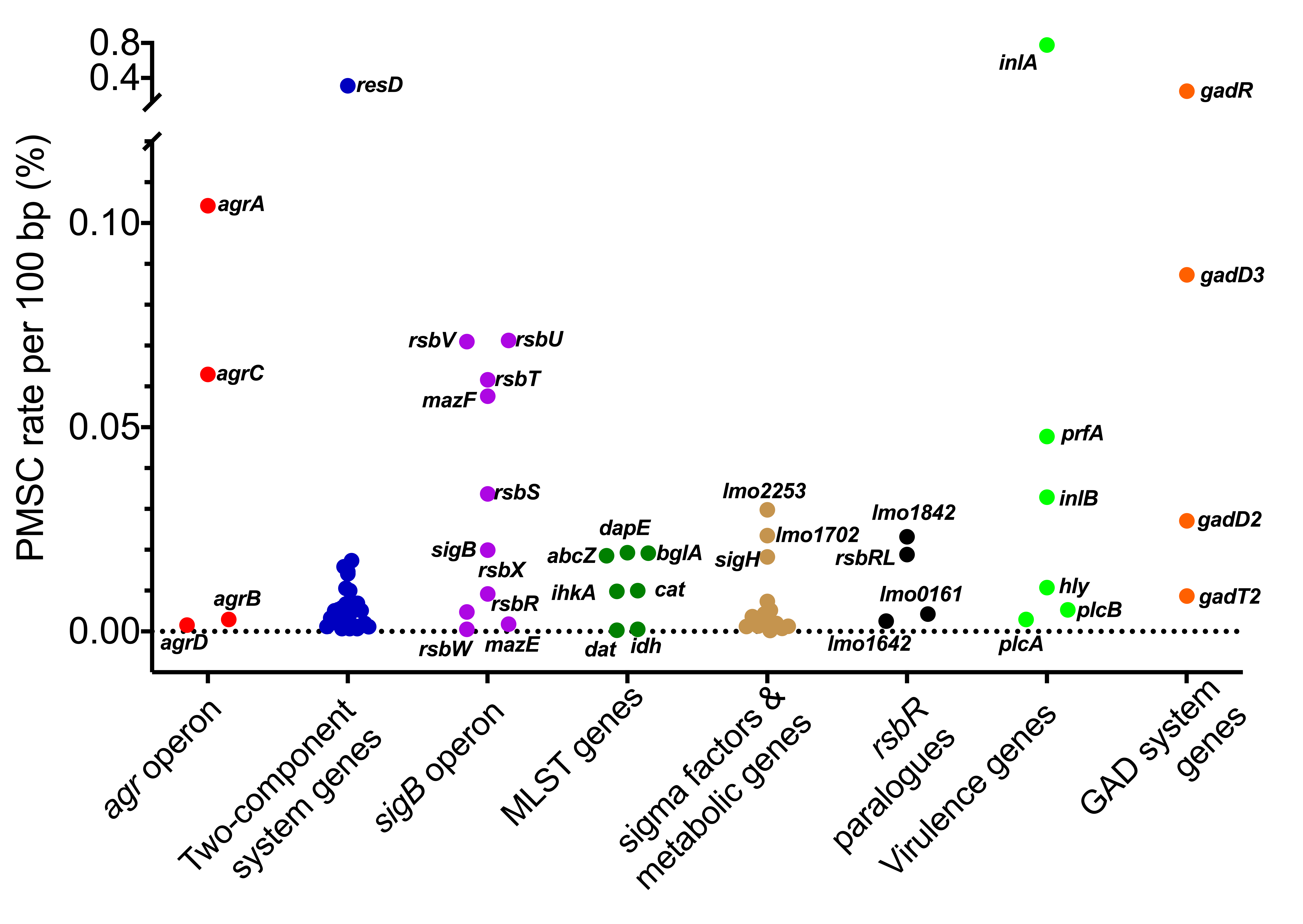
**

**Fig S5. High frequency of PMSCs was detected in the *gadR* coding sequence.**

*In silico* analysis was performed of 40,080 genomes of *L. monocytogenes* strains publicly available as previously described (1). PMSC rate normalized by 100 bp of the open reading frame length for genes comprising the *gadT2D2R* and *gadD3* are shown in comparison to those previously reported for: two-component systems genes, *sigB* operon, *mazEF*, MLST genes, sigma factors genes, metabolic genes, *rsbR1* paralogues, and virulence genes (1).

**STables**

Table S1 Plasmids and Primers used for creating mutants *L. monoctyogenes* EGD-e *gadR+* P*gadT2*_V1-V10.

| **Plasmid name** | **Source** |
| --- | --- |
| pJW21 pMAD::∆P*gadT2*_V1; Ery^r^; Amp^r^ | This study |
| pJW22 pMAD::P*gadT2*_V2; Ery^r^; Amp^r^ | This study |
| pJW23 pMAD::P*gadT2*_V3; Ery^r^; Amp^r^ | This study |
| pJW24 pMAD::P*gadT2*_V4; Ery^r^; Amp^r^ | This study |
| pJW25 pMAD::P*gadT2*_V5; Ery^r^; Amp^r^ | This study |
| pJW27 pMAD::P*gadT2*_V6; Ery^r^; Amp^r^ | This study |
| pJW28 pMAD::P*gadT2*_V7; Ery^r^; Amp^r^ | This study |
| pJW29 pMAD::P*gadT2*_V8; Ery^r^; Amp^r^ | This study |
| pJW30 pMAD::P*gadT2*_V9; Ery^r^; Amp^r^ | This study |
| pJW31 pMAD::P*gadT2*_V10; Ery^r^; Amp^r^ | This study |
| **Primer name** | **Primers sequence (5' - 3')** |
| JW21_SOE_up_F | ATATCCCGGGCGCTTCGTTCCAATCTATGA |
| JW21_SOE_up_R | CGAAAATACAACTAAACGAAGCGGAAGAGGGAGGATTTAATG |
| JW21_SOE_down_F | GCTTCGTTTAGTTGTATTTTCGC |
| JW21_SOE_down_R | ATATGGATCCGCCGTGTTTTTCGTACCACC |
| JW22_SOE_up_F | ATATGAATTCCGCTTCGTTCCAATCTATGA |
| JW22_SOE_up_R | CGAAAATACAACTAAACGAAGCCATTTTTAGTAAAAAACTCTTGCAC |
| JW23_SOE_up_R | CGAAAATACAACTAAACGAAGCAATGCTTATAGTTGTTCTATTCGC |
| JW24_SOE_down_F | GAACAACTATAAGCATTGGCTAAGAAAAAAACTGGAATAATTTTC |
| JW24_SOE_up_R | CAATGCTTATAGTTGTTCTATTCG |
| JW25_SOE_down_F | AACAAACGTGCTAATCTTATAC |
| JW25_SOE_up_R | GATTAGCACGTTTGTTAGTTTTTTTCTTAGCCAGGAAA |
| JW27_SOE_down_F | ACGTGCTAATCTTATACATGTTC |
| JW27_SOE_up_R | GTATAAGATTAGCACGTATTCCAGTTTTTTTCTTAGCCAG |
| JW28_SOE_down_F | CTCAAAAATTTTTCCTGACTGGAATAATTTTCGAAAACAAAC |
| JW28_SOE_up_R | CAGGAAAAATTTTTGAGACAATGC |
| JW29_SOE_down_F | GAAGAAATGGGAACATCTACC |
| JW29_SOE_up_R | GATGTTCCCATTTCTTCCGTAACTATTCATCACCGTTTG |
| JW31_SOE_down_F | GCTTTTCTCGTGTTAACTGAT |
| JW31_SOE_up_R | GTTAACACGAGAAAAGCTAAGTAATTGTTTGTTGGAACATG |

Table S2 Differentially expressed genes in strain EGD-e *gadR*^+^ vs wild type after acid treatment.

| **Gene** | **Fold change (log2)** | **Gene annotation** | **Gene** | **Fold change (log2)** | **Gene annotation** |
| --- | --- | --- | --- | --- | --- |
| ***lmo2362*** | 11.7 | amino acid antiporter | *lmos17* | -5.9 | rli27 |
| ***lmo2363*** | 9.3 | glutamate decarboxylase | *lmot54* | -5.2 | tRNA-Asp |
| ***lmo0265*** | 9.2 | succinyl-diaminopimelate desuccinylase | *lmo2507* | -4.9 | cell division protein FtsE |
| ***lmo2213*** | 8.8 | hypothetical protein | *lmo0394* | -4.9 | P60 protein |
| ***lmo0628*** | 8.8 | hypothetical protein | *lmo1671* | -4.9 | ABC transporter |
| ***lmo0913*** | 8.6 | succinate semialdehyde dehydrogenase | *lmot42* | -4.5 | tRNA-Thr |
| ***lmo2269*** | 8.6 | hypothetical protein | *lmo0186* | -4.4 | hypothetical protein |
| ***lmo2230*** | 8.2 | arsenate reductase | *lmot41* | -4.3 | tRNA-Glu |
| ***lmo0263*** | 8.2 | internalin H | *lmo1211* | -4.2 | hypothetical protein |
| ***lmo2602*** | 8.2 | hypothetical protein | *lmo2423* | -4.2 | hypothetical protein |
| ***lmo1694*** | 8.1 | CDP-abequose synthase | *lmot47* | -4.0 | tRNA-Cys |
| ***lmo1241*** | 8.0 | hypothetical protein | *lmo1216* | -3.9 | N-acetylmuramoyl-L-alanine amidase |
| ***lmo2673*** | 7.9 | hypothetical protein | *lmos28* | -3.9 | glmS |
| ***lmo0133*** | 7.9 | hypothetical protein | *lmot43* | -3.8 | tRNA-Tyr |
| ***lmo0019*** | 7.4 | hypothetical protein | *lmot48* | -3.8 | tRNA-Gly |
| ***lmo0043*** | 7.2 | arginine deiminase | *lmo0679* | -3.7 | flagellar biosynthesis protein FlhB |
| ***lmo2434*** | 7.2 | glutamate decarboxylase | *lmot30* | -3.7 | tRNA-Ala |
| ***lmo0880*** | 7.1 | wall associated protein precursor | *lmot44* | -3.7 | tRNA-Gln |
| ***lmo2158*** | 7.1 | hypothetical protein | *lmot28* | -3.5 | tRNA-Met |
| ***lmo2391*** | 6.9 | hypothetical protein | *lmo0678* | -3.5 | flagellar biosynthesis protein FliR |
| ***lmo2085*** | 6.8 | peptidoglycan binding protein | *lmo0272* | -3.4 | hypothetical protein |
| ***lmo2695*** | 6.7 | dihydroxyacetone kinase subunit DhaK | *lmo0676* | -3.4 | flagellar biosynthesis protein FliP |
| ***lmo0602*** | 6.7 | transcripitonal regulator | *lmo1447* | -3.4 | metal (zinc) transport protein(ABC transporter%2C ATP-binding protein) |
| ***lmo2387*** | 6.7 | hypothetical protein | *lmo0573* | -3.3 | hypothetical protein |
| ***lmo2748*** | 6.6 | hypothetical protein | *lmo2506* | -3.3 | cell division protein FtsX |
| ***lmo1830*** | 6.6 | short-chain dehydrogenase | *lmo0675* | -3.2 | hypothetical protein |
| *lmo2090* | 6.6 | argininosuccinate synthase | *lmo2048* | -3.2 | hypothetical protein |
| *lmo2210* | 6.5 | hypothetical protein | *lmo1707* | -3.1 | hypothetical protein |
| ***lmo2231*** | 6.5 | hypothetical protein | *lmo0469* | -3.1 | hypothetical protein |
| ***lmo2157*** | 6.4 | hypothetical protein | *lmo1840* | -3.1 | bifunctional pyrimidine regulatory protein PyrR uracil phosphoribosyltransferase |
| *lmo1113* | 6.4 | hypothetical protein | *lmo1210* | -3.0 | hypothetical protein |
| ***lmo2573*** | 6.4 | zinc-binding dehydrogenase | *lmo2241* | -3.0 | GntR family transcriptional regulator |
| ***lmo0134*** | 6.4 | hypothetical protein | *lmo0541* | -2.9 | ABC transporter substrate-binding protein |
| *lmo0445* | 6.3 | transcripitonal regulator | *lmo1396* | -2.9 | phosphatidylglycerophosphate synthase |
| ***lmo2067*** | 6.2 | bile acid hydrolase | *lmo0042* | -2.8 | DedA protein |
| ***lmo2603*** | 6.2 | hypothetical protein | *lmot22* | -2.8 | tRNA-Gly |
| *lmo1114* | 6.1 | hypothetical protein | *lmo0244* | -2.8 | 50S ribosomal protein L33 type II |
| ***lmo0994*** | 6.1 | hypothetical protein | *lmo0245* | -2.8 | preprotein translocase subunit SecE |
| ***lmo1140*** | 6.1 | hypothetical protein | *lmo2551* | -2.8 | transcription termination factor Rho |
| ***lmo0610*** | 6.0 | internalin | *lmo1298* | -2.7 | glutamine synthetase repressor |
| ***lmo0321*** | 6.0 | hypothetical protein | *lmo2505* | -2.7 | peptidoglycan lytic protein P45 |
| ***lmo2696*** | 6.0 | dihydroxyacetone kinase | *lmo1245* | -2.7 | hypothetical protein |
| ***lmo2572*** | 5.8 | dihydrofolate reductase subunit A | *lmo1839* | -2.7 | uracil permease |
| ***lmo0439*** | 5.7 | hypothetical protein | *lmot51* | -2.7 | tRNA-Trp |
| ***lmo0794*** | 5.6 | hypothetical protein | *lmo0695* | -2.7 | hypothetical protein |
| *lmo0997* | 5.6 | ATP-dependent protease | *lmo1498* | -2.6 | O-methyltransferase |
| ***lmo1433*** | 5.6 | glutathione reductase | *lmo2428* | -2.6 | cell division protein FtsW |
| ***lmo0169*** | 5.6 | glucose transporter | *lmo1500* | -2.6 | hypothetical protein |
| ***lmo2697*** | 5.6 | PTS mannose transporter subunit IIA | *lmo1073* | -2.6 | metal ABC transporter substrate-binding protein |
| ***lmo0722*** | 5.6 | pyruvate oxidase | *lmot49* | -2.6 | tRNA-Gln |
| *lmo2091* | 5.4 | argininosuccinate lyase | *lmot27* | -2.6 | tRNA-Ser |
| ***lmo2571*** | 5.3 | nicotinamidase | *lmo2433* | -2.6 | acetylesterase |
| ***lmo2132*** | 5.3 | hypothetical protein | *lmo1870* | -2.6 | alkaline phosphatase |
| ***lmo0539*** | 5.3 | tagatose 1%2C6-diphosphate aldolase | *lmo0246* | -2.6 | transcription antitermination protein NusG |
| ***lmo0953*** | 5.2 | hypothetical protein | *lmo2466* | -2.6 | hypothetical protein |
| *lmo1591* | 5.2 | N-acetyl-gamma-glutamyl-phosphate reductase | *lmo0277* | -2.6 | oxidoreductase |
| ***lmo0647*** | 5.2 | hypothetical protein | *lmo0696* | -2.5 | flagellar basal body rod modification protein |
| ***lmo2672*** | 5.1 | AraC family transcriptional regulator | *lmo2218* | -2.5 | hypothetical protein |
| ***lmo0596*** | 5.1 | hypothetical protein | *lmo1248* | -2.5 | hypothetical protein |
| ***lmo0654*** | 5.0 | hypothetical protein | *lmo0677* | -2.5 | flagellar biosynthesis protein FliQ |
| ***lmo0405*** | 5.0 | phosphate transporter | *lmo0130* | -2.5 | 5'-nucleotidase |
| ***lmo0784*** | 5.0 | PTS mannose transporter subunit IIB | *lmo0815* | -2.5 | transcriptional regulator |
| ***lmo0995*** | 4.9 | hypothetical protein | ***lmo1067*** | -2.5 | GTP-binding elongation factor |
| ***lmo2570*** | 4.9 | hypothetical protein | *lmo1937* | -2.5 | GTP-binding protein EngA |
| *lmo1734* | 4.8 | glutamate synthase large subunit | *lmo0816* | -2.4 | regulatory protein PaiA |
| ***lmo0783*** | 4.8 | PTS mannose transporter subunit IIB | *lmo0559* | -2.4 | hypothetical protein |
| ***lmo0670*** | 4.7 | hypothetical protein | *lmo2409* | -2.4 | hypothetical protein |
| ***lmo2205*** | 4.6 | phosphoglyceromutase | *lmo0219* | -2.4 | hypothetical protein |
| *lmo2495* | 4.6 | phosphate ABC transporter ATP-binding protein | *lmo2047* | -2.4 | 50S ribosomal protein L32 |
| ***lmo0911*** | 4.6 | hypothetical protein | *lmo0867* | -2.4 | hypothetical protein |
| *lmo0295* | 4.5 | FMN-containing NADPH-linked nitro/flavin reductase | *lmot50* | -2.4 | tRNA-His |
| *lmos81* | 4.5 | rli47 | *lmo0656* | -2.4 | hypothetical protein |
| ***lmo0274*** | 4.5 | hypothetical protein | *lmo2562* | -2.4 | hypothetical protein |
| ***lmo2494*** | 4.4 | PhoU family transcriptional regulator | *lmo1885* | -2.3 | xanthine phosphoribosyltransferase |
| ***lmo1789*** | 4.4 | hypothetical protein | *lmo1420* | -2.3 | UDP-N-acetylenolpyruvoylglucosamine reductase |
| *lmo2361* | 4.3 | hypothetical protein | *lmo2079* | -2.3 | hypothetical protein |
| *lmo1690* | 4.3 | hypothetical protein | *lmo0727* | -2.3 | glucosamine--fructose-6-phosphate aminotransferase |
| ***lmo0669*** | 4.2 | oxidoreductase | *lmo0465* | -2.3 | hypothetical protein |
| ***lmo0515*** | 4.2 | hypothetical protein | *lmo2337* | -2.3 | DeoR family transcriptional regulator |
| ***lmo1526*** | 4.2 | hypothetical protein | *lmot20* | -2.3 | tRNA-Asn |
| *lmo1788* | 4.2 | transcriptional regulator | *lmo1446* | -2.2 | metal (zinc) transport protein (ABC transporter%2C permease) |
| ***lmo0554*** | 4.2 | NADH-dependent butanol dehydrogenase | *lmo0198* | -2.2 | bifunctional N-acetylglucosamine-1-phosphate uridyltransferase/glucosamine-1-phosphate acetyltransferase |
| *lmo0211* | 4.1 | 50S ribosomal protein L25 | *lmo0948* | -2.2 | transcriptional regulator |
| ***lmo0433*** | 4.1 | internalin A | *lmo0847* | -2.2 | glutamine ABC transporter |
| *lmo2399* | 4.1 | hypothetical protein | *lmo1228* | -2.2 | ribonuclease HIII |
| ***lmo1790*** | 4.1 | hypothetical protein | *lmo1616* | -2.2 | hypothetical protein |
| ***lmo0782*** | 4.0 | PTS mannose transporter subunit IIC | *lmo2063* | -2.2 | hypothetical protein |
| *lmo2567* | 4.0 | hypothetical protein | *lmo1419* | -2.2 | hypothetical protein |
| ***lmo2724*** | 4.0 | hypothetical protein | *lmo1479* | -2.2 | GTP-binding protein LepA |
| *lmo1293* | 4.0 | glycerol-3-phosphate dehydrogenase | *lmo2186* | -2.2 | hypothetical protein |
| ***lmo2174*** | 4.0 | hypothetical protein | *lmo1615* | -2.2 | tRNA (guanine-N(7)-)-methyltransferase |
| ***lmo2156*** | 4.0 | hypothetical protein | *lmo1007* | -2.1 | hypothetical protein |
| ***lmo0629*** | 3.9 | hypothetical protein | *lmo0361* | -2.1 | membrane protein |
| ***lmo0796*** | 3.9 | hypothetical protein | *lmo1431* | -2.1 | ABC transporter ATP-binding protein |
| *lmo2568* | 3.8 | hypothetical protein | *lmo2352* | -2.1 | LysR family transcriptional regulator |
| ***lmo1580*** | 3.7 | hypothetical protein | ***lmo1014*** | -2.1 | glycine/betaine ABC transporter ATP-binding protein |
| ***lmo2511*** | 3.7 | hypothetical protein | *lmo1714* | -2.1 | hypothetical protein |
| ***lmo2398*** | 3.7 | hypothetical protein | *lmo1749* | -2.1 | shikimate kinase |
| ***lmo0292*** | 3.7 | heat-shock protein htrA serine protease | *lmo2563* | -2.1 | hypothetical protein |
| *lmo2453* | 3.6 | epoxide hydrolase | *lmo0691* | -2.1 | chemotaxis response regulator CheY |
| ***lmo2454*** | 3.6 | hypothetical protein | *lmo1826* | -2.1 | DNA-directed RNA polymerase subunit omega |
| *lmo2206* | 3.6 | Clp protease subunit B | *lmot29* | -2.1 | tRNA-Met |
| *lmo0325* | 3.6 | transcriptional regulator | *lmo1499* | -2.0 | hypothetical protein |
| ***lmo0170*** | 3.6 | hypothetical protein | *lmo1496* | -2.0 | transcription elongation factor GreA |
| ***lmo0655*** | 3.6 | phosphoprotein phosphatase | *lmo0199* | -2.0 | ribose-phosphate pyrophosphokinase |
| *lmo0759* | 3.5 | hypothetical protein | *lmo2197* | -2.0 | hypothetical protein |
| *lmo2250* | 3.5 | amino acid ABC transporter permease | *lmo2504* | -2.0 | cell wall-binding protein |
| *lmo2006* | 3.5 | acetolactate synthase | *lmo2240* | -2.0 | ABC transporter ATP-binding protein |
| *lmo0760* | 3.5 | hypothetical protein | *lmo0470* | -2.0 | hypothetical protein |
| ***lmo2484*** | 3.5 | hypothetical protein | *lmo1440* | -2.0 | hypothetical protein |
| *lmo2436* | 3.4 | transcription antiterminator | *lmo2754* | -2.0 | D-alanyl-D-alanine carboxypeptidase |
| ***lmo0937*** | 3.4 | hypothetical protein | *lmo0514* | -2.0 | internalin |
| ***lmo1539*** | 3.4 | glycerol transporter | *lmo1492* | -2.0 | hypothetical protein |
| *lmo2714* | 3.4 | pepdidoglycan bound protein | *lmo2550* | -2.0 | glycosyl transferase |
| ***lmo0870*** | 3.4 | hypothetical protein | *lmo1328* | -2.0 | tRNA pseudouridine synthase B |
| ***lmo1068*** | 3.3 | hypothetical protein | *lmot52* | -2.0 | tRNA-Tyr |
| *lmo0229* | 3.3 | CtsR family transcriptional regulator | *lmo0697* | -2.0 | flagellar hook protein FlgE |
| ***lmo2670*** | 3.3 | hypothetical protein | *lmo0256* | -2.0 | hypothetical protein |
| ***lmo1992*** | 3.3 | alpha-acetolactate decarboxylase | *lmot56* | -1.9 | tRNA-Val |
| ***lmo0579*** | 3.3 | hypothetical protein | *lmo1752* | -1.9 | hypothetical protein |
| ***lmo2674*** | 3.3 | ribose-5-phosphate isomerase B | *lmo2690* | -1.9 | TetR family transcriptional regulator |
| *lmo2360* | 3.2 | transmembrane protein | *lmot57* | -1.9 | tRNA-Glu |
| *lmo1424* | 3.2 | manganese transporter | *lmo0882* | -1.9 | hypothetical protein |
| *lmo1917* | 3.2 | pyruvate formate-lyase | *lmo2277* | -1.9 | hypothetical protein |
| ***lmo2386*** | 3.2 | hypothetical protein | *lmo0436* | -1.9 | hypothetical protein |
| ***lmo0231*** | 3.2 | ATP:guanido phosphotransferase | *lmot21* | -1.9 | tRNA-Ile |
| ***lmo2463*** | 3.2 | multidrug transporter | *lmo1074* | -1.9 | teichoic acid translocation permease TagG |
| *lmo0584* | 3.2 | hypothetical protein | *lmo1603* | -1.9 | aminopeptidase |
| *lmo0095* | 3.1 | hypothetical protein | *lmo2254* | -1.9 | hypothetical protein |
| ***lmo2358*** | 3.1 | N-acetylglucosamine-6-phosphate isomerase | *lmo0176* | -1.8 | glucose transporter |
| *lmo0761* | 3.1 | hypothetical protein | *lmo0947* | -1.8 | hypothetical protein |
| ***lmo2191*** | 3.1 | ArsC family transcriptional regulator | *lmo2857* | -1.8 | hypothetical protein |
| ***lmo0230*** | 3.1 | hypothetical protein | *lmo1333* | -1.8 | hypothetical protein |
| *lmo0819* | 3.0 | hypothetical protein | *lmo1661* | -1.8 | hypothetical protein |
| *lmo0291* | 3.0 | hypothetical protein | *lmo0185* | -1.8 | hypothetical protein |
| *lmo2251* | 3.0 | amino acid ABC transporter ATP-binding protein | *lmo2408* | -1.8 | repressor protein |
| *lmos50* | 2.9 | rli42 | *lmo0837* | -1.8 | hypothetical protein |
| *lmo0800* | 2.9 | hypothetical protein | *lmo1697* | -1.8 | hypothetical protein |
| ***lmo0869*** | 2.9 | hypothetical protein | *lmot19* | -1.8 | tRNA-Ser |
| ***lmo0434*** | 2.9 | internalin B | *lmot10* | -1.8 | tRNA-Ala |
| ***lmo0406*** | 2.9 | hypothetical protein | *lmo1952* | -1.8 | diaminopimelate decarboxylase |
| *lmo2189* | 2.9 | competence protein CoiA | *lmo1957* | -1.8 | ferrichrome ABC transporter permease |
| *lmo0375* | 2.9 | hypothetical protein | *lmo1260* | -1.8 | gamma-glutamyl kinase |
| *lmo0925* | 2.9 | hypothetical protein | *lmo1677* | -1.8 | 1%2C4-dihydroxy-2-naphthoate octaprenyltransferase |
| *lmo0660* | 2.9 | transposase | *lmo2061* | -1.8 | hypothetical protein |
| ***lmo1375*** | 2.9 | aminotripeptidase | *lmo2058* | -1.8 | heme O oxygenase |
| ***lmo0580*** | 2.9 | hypothetical protein | *lmo2539* | -1.7 | serine hydroxymethyltransferase |
| *lmo2575* | 2.9 | cation transporter | *lmo2559* | -1.7 | CTP synthetase |
| *lmo1138* | 2.8 | ATP-dependent Clp protease proteolytic subunit | *lmo0008* | -1.7 | cardiolipin synthase |
| *lmo0904* | 2.8 | hypothetical protein | *lmo0362* | -1.7 | hypothetical protein |
| ***lmo2485*** | 2.8 | hypothetical protein | *lmo0785* | -1.7 | transcriptional regulator |
| *lmo2784* | 2.8 | transcriptional antiterminator | *lmo0718* | -1.7 | hypothetical protein |
| *lmo0923* | 2.8 | ABC transporter ATP-binding protein | *lmo1071* | -1.7 | cell division protein FtsW |
| *lmos27* | 2.8 | rli33 | *lmo2062* | -1.7 | copper transporter |
| *lmo0924* | 2.7 | - | *lmo1585* | -1.7 | peptidase |
| *lmo2005* | 2.7 | oxidoreductase | *lmo1635* | -1.7 | hypothetical protein |
| ***lmo0781*** | 2.7 | PTS mannose transporter subunit IID | *lmo0793* | -1.7 | hypothetical protein |
| ***lmo0956*** | 2.7 | N-acetylglucosamine-6P-phosphate deacetylase | *lmo1994* | -1.7 | LacI family transcriptional regulator |
| ***lmo0524*** | 2.7 | sulfate transporter | *lmo1810* | -1.7 | fatty acid biosynthesis transcriptional regulator |
| *lmo2828* | 2.7 | hypothetical protein | *lmot23* | -1.7 | tRNA-His |
| *lmo2242* | 2.7 | O6-methylguanine-DNA methyltransferase | *lmo1884* | -1.7 | xanthine permease |
| *lmo1684* | 2.7 | glycerate dehydrogenase | *lmo1996* | -1.7 | DeoR family transcriptional regulator |
| ***lmo1428*** | 2.6 | glycine/betaine ABC transporter ATP-binding protein | *lmo2526* | -1.7 | UDP-N-acetylglucosamine 1-carboxyvinyltransferase |
| ***lmo1295*** | 2.6 | host factor-1 protein | *lmo0294* | -1.7 | LysR family transcriptional regulator |
| ***lmo2190*** | 2.6 | adaptor protein | *lmo0790* | -1.7 | transcriptional regulator |
| *lmo0943* | 2.6 | non-heme iron-binding ferritin | *lmo0665* | -1.7 | hypothetical protein |
| *lmo0377* | 2.6 | hypothetical protein | *lmo1625* | -1.7 | transporter |
| ***lmo2468*** | 2.5 | ATP-dependent Clp protease proteolytic subunit | *lmo2465* | -1.7 | hypothetical protein |
| *lmo2792* | 2.5 | hypothetical protein | *lmo1866* | -1.7 | hypothetical protein |
| *lmo2487* | 2.5 | hypothetical protein | *lmo1751* | -1.7 | hypothetical protein |
| *lmo2357* | 2.5 | hypothetical protein | *lmo2591* | -1.7 | N-acetylmuramoyl-L-alanine amidase |
| ***lmo1340*** | 2.5 | hypothetical protein | *lmo0694* | -1.7 | hypothetical protein |
| *lmo1423* | 2.5 | hypothetical protein | *lmo1548* | -1.6 | rod shape-determining protein MreB |
| *lmo0266* | 2.5 | transcriptional regulator | *lmo0193* | -1.6 | hypothetical protein |
| ***lmo1601*** | 2.5 | general stress protein | *lmo1024* | -1.6 | hypothetical protein |
| *lmo1849* | 2.5 | metal ABC transporter ATP-binding protein | *lmo0597* | -1.6 | Crp/Fnr family transcriptional regulator |
| *lmo0209* | 2.5 | hypothetical protein | *lmo0510* | -1.6 | hypothetical protein |
| *lmo1966* | 2.5 | hypothetical protein | *lmo1545* | -1.6 | septum formation inhibitor MinC |
| *lmo0926* | 2.5 | TetR family transcriptional regulator | *lmo2040* | -1.6 | cell division protein FtsL |
| ***lmo1602*** | 2.4 | hypothetical protein | *lmo1568* | -1.6 | hypothetical protein |
| *lmo2366* | 2.4 | DeoR family transcriptional regulator | *lmo1776* | -1.6 | hypothetical protein |
| *lmo0114* | 2.4 | repressor C1 | *lmo0475* | -1.6 | hypothetical protein |
| *lmo0604* | 2.4 | hypothetical protein | *lmo0009* | -1.6 | spermidine acetyltransferase |
| *lmo0471* | 2.4 | hypothetical protein | *lmo2045* | -1.6 | hypothetical protein |
| *lmo0339* | 2.4 | hypothetical protein | *lmo0959* | -1.6 | undacaprenyl-phosphate N-acetylglucosaminyltransferase |
| ***lmo1538*** | 2.4 | glycerol kinase | *lmo0533* | -1.6 | hypothetical protein |
| *lmo2830* | 2.4 | thioredoxin | *lmo0806* | -1.6 | transcriptional regulator |
| *lmo2679* | 2.4 | histidine kinase | *lmo0883* | -1.6 | hypothetical protein |
| *lmo0341* | 2.4 | hypothetical protein | *lmo1537* | -1.6 | GTPase ObgE |
| ***lmo1427*** | 2.3 | glycine/betaine ABC transporter permease | *lmo1567* | -1.6 | citrate synthase |
| *lmo0113* | 2.3 | hypothetical protein | *lmo2758* | -1.6 | inosine-monophosphate dehydrogenase |
| *lmo2739* | 2.3 | NAD-dependent deacetylase | *lmo0692* | -1.6 | two-component sensor histidine kinase CheA |
| *lmo2680* | 2.3 | potassium-transporting ATPase subunit C | *lmo1309* | -1.6 | hypothetical protein |
| *lmo1518* | 2.3 | hypothetical protein | *lmo2128* | -1.6 | LacI family transcriptional regulator |
| *lmo0496* | 2.3 | hypothetical protein | *lmo0418* | -1.6 | hypothetical protein |
| ***lmo1421*** | 2.3 | glycine/betaine ABC transporter ATP-binding protein | *lmo1515* | -1.6 | hypothetical protein |
| *lmo0758* | 2.3 | hypothetical protein | *lmo1360* | -1.6 | bifunctional 5%2C10-methylene-tetrahydrofolate dehydrogenase/ 5%2C10-methylene-tetrahydrofolate cyclohydrolase |
| *lmo2406* | 2.3 | hypothetical protein | *lmo2538* | -1.5 | uracil phosphoribosyltransferase |
| *lmo1739* | 2.3 | amino acid ABC transporter ATP-binding protein | *lmo1078* | -1.5 | UDP-glucose pyrophosphorylase |
| *lmo1018* | 2.2 | copper homeostasis protein CutC | *lmo2549* | -1.5 | wall teichoic acid glycosylation protein GtcA |
| *lmo0813* | 2.2 | fructokinase | *lmo2046* | -1.5 | 2-dehydropantoate 2-reductase |
| *lmo0818* | 2.2 | cation-transporting ATPase | *lmo1891* | -1.5 | Holliday junction-specific endonuclease |
| *lmo0606* | 2.2 | MarR family transcriptional regulator | *lmo0571* | -1.5 | methyltransferase |
| ***lmo1426*** | 2.2 | glycine/betaine ABC transporter substrate-binding protein | *lmo1685* | -1.5 | glutamate-1-semialdehyde aminotransferase |
| *lmo0955* | 2.2 | hypothetical protein | *lmo0537* | -1.5 | allantoate amidohydrolase |
| *lmo0964* | 2.2 | hypothetical protein | *lmo1626* | -1.5 | hypothetical protein |
| *lmo2574* | 2.2 | hypothetical protein | *lmo0132* | -1.5 | inosine 5-monophosphate dehydrogenase |
| *lmo0954* | 2.2 | hypothetical protein | *lmo2811* | -1.5 | tRNA modification GTPase TrmE |
| *lmo1740* | 2.1 | amino acid ABC transporter permease | *lmo2842* | -1.5 | LacI family transcriptional regulator |
| *lmo2173* | 2.1 | sigma-54-dependent transcriptional regulator | *lmo0253* | -1.5 | penicillinase antirepressor |
| *lmo2742* | 2.1 | hypothetical protein | *lmo0218* | -1.5 | hypothetical protein |
| *lmo0340* | 2.1 | hypothetical protein | *lmo0482* | -1.5 | ribosomal RNA large subunit methyltransferase N |
| *lmo1964* | 2.1 | ABC transporter ATP-binding protein | *lmo1555* | -1.5 | uroporphyrinogen-III synthase |
| *lmo0078* | 2.1 | phosphoglycerate dehydrogenase | *lmo2547* | -1.5 | homoserine dehydrogenase |
| *lmo2107* | 2.1 | DeoR family transcriptional regulator | *lmo1530* | -1.5 | queuine tRNA-ribosyltransferase |
| ***lmo1425*** | 2.1 | glycine/betaine ABC transporter permease | *lmo0509* | -1.5 | phosphoribosyl pyrophosphate synthetase |
| *lmo1967* | 2.1 | toxic ion resistance protein | *lmo1384* | -1.5 | hypothetical protein |
| *lmo2373* | 2.0 | PTS beta-glucoside transporter subunit IIB | *lmo2052* | -1.4 | phosphopantetheine adenylyltransferase |
| ***lmo0232*** | 2.0 | endopeptidase Clp ATP-binding chain C | *lmo0511* | -1.4 | hypothetical protein |
| *lmo1792* | 2.0 | tRNA (guanine-N(1)-)-methyltransferase | *lmo0998* | -1.4 | hypothetical protein |
| *lmo2407* | 2.0 | hypothetical protein | *lmo1491* | -1.4 | GTP-binding protein |
| *lmo2709* | 2.0 | hypothetical protein | *lmo2561* | -1.4 | arginyl-tRNA synthetase |
| *lmo0081* | 2.0 | hypothetical protein | *lmo2537* | -1.4 | UDP-N-acetylglucosamine 2-epimerase |
| *lmo2593* | 2.0 | MerR family transcriptional regulator | *lmo2779* | -1.4 | GTP-binding protein EngD |
| *lmo2486* | 2.0 | hypothetical protein | *lmo1296* | -1.4 | hypothetical protein |
| ***lmo1261*** | 2.0 | hypothetical protein | *lmo0582* | -1.4 | invasion associated secreted endopeptidase |
| *lmo1791* | 2.0 | hypothetical protein | *lmo1030* | -1.4 | LacI family transcriptional regulator |
| *lmo2252* | 2.0 | aspartate aminotransferase | *lmo1827* | -1.4 | guanylate kinase |
| *lmo0822* | 1.9 | transcriptional regulator | *lmo1389* | -1.4 | sugar ABC transporter ATP-binding protein |
| *lmo2177* | 1.9 | hypothetical protein | *lmo1348* | -1.4 | glycine cleavage system aminomethyltransferase T |
| *lmo2713* | 1.9 | internalin | *lmo1882* | -1.4 | 30S ribosomal protein S14 |
| *lmo0932* | 1.9 | hypothetical protein | *lmo2569* | -1.4 | peptide ABC transporter substrate-binding protein |
| *lmo0788* | 1.9 | hypothetical protein | *lmo0789* | -1.4 | hypothetical protein |
| *lmo0642* | 1.9 | hypothetical protein | *lmo0666* | -1.4 | hypothetical protein |
| *lmo0905* | 1.9 | hypothetical protein | *lmo0288* | -1.4 | two-component sensor histidine kinase |
| *lmo2743* | 1.9 | translaldolase | *lmo1096* | -1.4 | GMP synthase |
| *lmo1738* | 1.9 | amino acid ABC transporter substrate-binding protein | *lmo2710* | -1.4 | hypothetical protein |
| *lmo0805* | 1.9 | hypothetical protein | *lmo2249* | -1.4 | low-affinity inorganic phosphate transporter |
| *lmo1257* | 1.9 | hypothetical protein | ***lmo0723*** | -1.4 | metyl-accepting chemotaxis protein |
| ***lmo1704*** | 1.9 | hypothetical protein | *lmo0707* | -1.4 | flagellar capping protein FliD |
| *lmo2258* | 1.9 | hypothetical protein | *lmo1445* | -1.3 | ZurR family transcriptional regulator |
| *lmo1618* | 1.8 | MarR family transcriptional regulator | *lmo1026* | -1.3 | LytR protein |
| ***lmo1606*** | 1.8 | DNA translocase | *lmo1497* | -1.3 | uridine kinase |
| *lmo2338* | 1.8 | aminopeptidase | *lmo1748* | -1.3 | hypothetical protein |
| *lmo1137* | 1.8 | hypothetical protein | *lmo1259* | -1.3 | gamma-glutamyl phosphate reductase |
| *lmo0871* | 1.8 | hypothetical protein | *lmo2248* | -1.3 | hypothetical protein |
| ***lmo2460*** | 1.8 | transcriptional regulator | *lmo1520* | -1.3 | histidyl-tRNA synthetase |
| *lmo0850* | 1.8 | hypothetical protein | *lmo0152* | -1.3 | peptide ABC transporter substrate-binding protein |
| *lmo2200* | 1.8 | MarR family transcriptional regulator | *lmo1355* | -1.3 | elongation factor P |
| *lmo2478* | 1.8 | thioredoxin reductase | *lmo1689* | -1.3 | A/G-specific adenine glycosylase |
| *lmo0607* | 1.8 | ABC transporter ATP-binding protein | ***lmo0724*** | -1.3 | hypothetical protein |
| *lmo0720* | 1.8 | hypothetical protein | *lmo0927* | -1.3 | hypothetical protein |
| *lmo1258* | 1.8 | hypothetical protein | *lmot58* | -1.3 | tRNA-Ser |
| *lmo1102* | 1.8 | cadmium efflux system accessory protein | *lmo1879* | -1.3 | cold-shock protein |
| *lmo0719* | 1.8 | hypothetical protein | *lmos03* | -1.3 | SAM |
| *lmo0047* | 1.8 | hypothetical protein | *lmo2053* | -1.3 | hypothetical protein |
| *lmo2069* | 1.8 | co-chaperonin GroES | *lmo0826* | -1.3 | transporter |
| *lmo1407* | 1.7 | pyruvate-formate lyase activating enzyme | *lmo0645* | -1.3 | amino acid transporter |
| *lmo2675* | 1.7 | hypothetical protein | *lmo0988* | -1.3 | peptide chain release factor 3 |
| *lmo1475* | 1.7 | heat-inducible transcription repressor | *lmo0474* | -1.3 | hypothetical protein |
| *lmo0612* | 1.7 | MarR family transcriptional evidence | *lmo1981* | -1.3 | hypothetical protein |
| *lmo1848* | 1.7 | metal ABC transporter permease | *lmo0945* | -1.3 | competence protein ComEC |
| *lmo1220* | 1.7 | hypothetical protein | *lmo1809* | -1.3 | glycerol-3-phosphate acyltransferase PlsX |
| *lmo0578* | 1.7 | hypothetical protein | *lmo1294* | -1.3 | tRNA delta(2)-isopentenylpyrophosphate transferase |
| ***lmo1454*** | 1.7 | RNA polymerase sigma factor RpoD | *lmo0981* | -1.3 | transporter |
| *lmo2031* | 1.6 | hypothetical protein | *lmo2051* | -1.3 | hypothetical protein |
| *lmo2030* | 1.6 | hypothetical protein | *lmo1118* | -1.3 | hypothetical protein |
| *lmo2439* | 1.6 | hypothetical protein | *lmo2802* | -1.3 | 16S rRNA methyltransferase GidB |
| *lmo1302* | 1.6 | LexA family transcriptional regulator | *lmo1321* | -1.3 | hypothetical protein |
| ***lmo0210*** | 1.6 | L-lactate dehydrogenase | *lmo2516* | -1.3 | hypothetical protein |
| *lmo0733* | 1.6 | transcriptional regulator | *lmo0903* | -1.3 | hypothetical protein |
| *lmo2108* | 1.6 | N-acetylglucosamine-6-phosphate deacetylase | *lmo0484* | -1.3 | heme-degrading monooxygenase IsdG |
| *lmo0909* | 1.6 | GntR family transcriptional regulator | *lmos79* | -1.3 | rli46 |
| ***lmo0957*** | 1.6 | glucosamine-6-phosphate isomerase | *lmo1892* | -1.3 | penicillin-binding protein 2A |
| *lmo2370* | 1.6 | aminotransferase | ***lmo1702*** | -1.3 | glutathione transferase |
| *lmo2199* | 1.6 | hypothetical protein | *lmo2050* | -1.3 | excinuclease ABC subunit A |
| *lmo2112* | 1.6 | hypothetical protein | *lmo2552* | -1.3 | UDP-N-acetylglucosamine 1-carboxyvinyltransferase |
| *lmo1474* | 1.5 | heat shock protein GrpE | *lmo1483* | -1.3 | competence protein ComEB |
| *lmo0829* | 1.5 | pyruvate-flavodoxin oxidoreductase | *lmo1485* | -1.3 | hypothetical protein |
| ***lmo0200*** | 1.5 | listeriolysin positive regulatory protein | *lmo0192* | -1.3 | PurR family transcriptional regulator |
| *lmo2785* | 1.5 | catalase | *lmo1828* | -1.3 | hypothetical protein |
| *lmo2028* | 1.5 | hypothetical protein | *lmo2831* | -1.3 | phosphoglucomutase |
| *lmo0082* | 1.5 | hypothetical protein | *lmo1490* | -1.3 | shikimate 5-dehydrogenase |
| ***lmo0555*** | 1.5 | di-tripeptide transporter | *lmo0055* | -1.3 | adenylosuccinate synthetase |
| *lmo0804* | 1.5 | hypothetical protein | *lmo0831* | -1.3 | hypothetical protein |
| *lmo2592* | 1.5 | aldo/keto reductase | *lmo2208* | -1.2 | hypothetical protein |
| *lmo0553* | 1.5 | hypothetical protein | *lmo0763* | -1.2 | hypothetical protein |
| *lmo2271* | 1.5 | hypothetical protein | *lmo1353* | -1.2 | hypothetical protein |
| ***lmo1432*** | 1.5 | hypothetical protein | *lmo0717* | -1.2 | transglycosylase |
| *lmo0974* | 1.4 | D-alanine--poly(phosphoribitol) ligase subunit 1 | *lmot46* | -1.2 | tRNA-Leu |
| *lmo0257* | 1.4 | hypothetical protein | *lmo0693* | -1.2 | flagellar motor switch protein FliY |
| *lmo1301* | 1.4 | hypothetical protein | *lmo1313* | -1.2 | uridylate kinase |
| ***lmo2462*** | 1.4 | dipeptidase | *lmo1556* | -1.2 | porphobilinogen deaminase |
| *lmo0625* | 1.4 | hypothetical protein | *lmo2744* | -1.2 | Crp/Fnr family transcriptional regulator |
| *lmo1381* | 1.4 | acylphosphatase | *lmo0908* | -1.2 | hypothetical protein |
| ***lmo1933*** | 1.4 | GTP cyclohydrolase I | *lmo0787* | -1.2 | amino acid transporter |
| *lmo0577* | 1.4 | hypothetical protein | *lmo1417* | -1.2 | hypothetical protein |
| *lmo1285* | 1.4 | hypothetical protein | *lmo0177* | -1.2 | methionyl-tRNA synthetase |
| *lmo1189* | 1.4 | transcriptional regulator | *lmo1639* | -1.2 | DNA-3-methyladenine glycosidase |
| *lmo0798* | 1.4 | lysine-specific permease | *lmo1456* | -1.2 | hypothetical protein |
| *lmo2068* | 1.4 | molecular chaperone GroEL | *lmo2491* | -1.2 | hypothetical protein |
| *lmo2176* | 1.4 | TetR family transcriptional regulator | *lmo0762* | -1.2 | ATP/GTP-binding protein |
| *lmo1460* | 1.4 | DNA repair protein RecO | *lmo1308* | -1.2 | hypothetical protein |
| *lmo2029* | 1.4 | hypothetical protein | *lmo1664* | -1.2 | S-adenosylmethionine synthetase |
| *lmo1617* | 1.4 | multidrug transporter | *lmo0811* | -1.2 | carbonic anhydrase |
| *lmo0267* | 1.4 | hypothetical protein | ***lmo0222*** | -1.2 | heat shock protein 33 |
| *lmo0080* | 1.4 | hypothetical protein | *lmo2378* | -1.2 | monovalent cation/H+ antiporter subunit A |
| ***lmo1713*** | 1.3 | rod shape-determining protein MreB | *lmo0404* | -1.2 | hypothetical protein |
| *lmo2745* | 1.3 | ABC transporter ATP-binding protein | ***lmo0221*** | -1.2 | pantothenate kinase |
| *lmo2676* | 1.3 | DNA polymerase IV | *lmo1338* | -1.2 | hypothetical protein |
| *lmo2707* | 1.3 | hypothetical protein | *lmo0419* | -1.2 | hypothetical protein |
| *lmo0466* | 1.3 | hypothetical protein | *lmo1699* | -1.2 | chemotaxis protein |
| *lmo2389* | 1.3 | NADH dehydrogenase | *lmo2855* | -1.2 | ribonuclease P |
| *lmo1244* | 1.3 | phosphoglycerate mutase | *lmo0077* | -1.2 | hypothetical protein |
| *lmo1708* | 1.3 | aminoglycoside N3'-acetyltransferase | *lmo1908* | -1.2 | hypothetical protein |
| *lmo0131* | 1.3 | hypothetical protein | *lmo1744* | -1.2 | hypothetical protein |
| *lmo2054* | 1.2 | hypothetical protein | *lmo2519* | -1.2 | teichoic acid linkage unit synthesis protein |
| *lmo2684* | 1.2 | PTS cellbiose transporter subunit IIC | *lmo2039* | -1.2 | penicillin-binding protein 2B |
| *lmo2685* | 1.2 | PTS cellbiose transporter subunit IIA | *lmos44* | -1.2 | T-box |
| *lmo0208* | 1.2 | hypothetical protein | *lmo2202* | -1.2 | 3-oxoacyl-ACP synthase |
| *lmo0930* | 1.2 | hypothetical protein | *lmo2041* | -1.2 | S-adenosyl-methyltransferase MraW |
| *lmo0352* | 1.2 | DeoR family transcriptional regulator | *lmo0370* | -1.2 | hypothetical protein |
| *lmo0521* | 1.2 | 6-phospho-beta-glucosidase | *lmo1600* | -1.2 | bifunctional 3-deoxy-7-phosphoheptulonate synthase/chorismate mutase |
| *lmo2204* | 1.2 | hypothetical protein | *lmo1557* | -1.1 | glutamyl-tRNA reductase |
| *lmo2701* | 1.2 | hypothetical protein | *lmo1090* | -1.1 | glycosyltransferase |
| *lmo2472* | 1.2 | hypothetical protein | *lmo0452* | -1.1 | hypothetical protein |
| ***lmo0407*** | 1.2 | hypothetical protein | *lmo0777* | -1.1 | hypothetical protein |
| *lmo2728* | 1.2 | MerR family transcriptional regulator | *lmo0158* | -1.1 | hypothetical protein |
| *lmo1569* | 1.2 | FxsA | *lmo0698* | -1.1 | flagellar motor switch protein |
| *lmo0906* | 1.2 | glutathione reductase | *lmo0497* | -1.1 | sugar transferase |
| ***lmo0893*** | 1.2 | anti-anti-sigma factor (antagonist of RsbW) | *lmo1638* | -1.1 | hypothetical protein |
| *lmos35* | 1.2 | rli53 | *lmo1865* | -1.1 | hypothetical protein |
| *lmo2055* | 1.2 | hypothetical protein | *lmo1808* | -1.1 | ACP S-malonyltransferase |
| *lmo1847* | 1.2 | metal ABC transporter | *lmo1089* | -1.1 | glycerol-3-phosphate cytidylyltransferase |
| *lmo1408* | 1.2 | hypothetical protein | *lmo1076* | -1.1 | autolysin |
| ***lmo0648*** | 1.2 | hypothetical protein | *lmo0440* | -1.1 | hypothetical protein |
| *lmo1579* | 1.2 | alanine dehydrogenase | *lmo2244* | -1.1 | ribosomal large subunit pseudouridine synthase |
| *lmo2706* | 1.2 | hypothetical protein | *lmo1536* | -1.1 | prephenate dehydratase |
| *lmo1894* | 1.1 | endonuclease III (DNA repair) | *lmo0135* | -1.1 | peptide ABC transporter substrate-binding protein |
| *lmo0858* | 1.1 | LacI family transcriptional regulator | *lmo0664* | -1.1 | acetyl transferase |
| ***lmo1300*** | 1.1 | arsenic transporter | ***lmo0527*** | -1.1 | transmembrane protein |
| *lmo0812* | 1.1 | hypothetical protein | *lmo1235* | -1.1 | aspartate kinase |
| *lmo0615* | 1.1 | hypothetical protein | *lmo1489* | -1.1 | hypothetical protein |
| *lmo1895* | 1.1 | chromosome replication initiation protein | *lmo2038* | -1.1 | UDP-N-acetylmuramoylalanyl-D-glutamate--2%2C6-diaminopimelate ligase |
| *lmo0271* | 1.1 | phospho-beta-glucosidase | *lmo2492* | -1.1 | hypothetical protein |
| *lmo0797* | 1.1 | hypothetical protein | *lmo1558* | -1.1 | GTP-binding protein EngB |
| *lmo0910* | 1.1 | hypothetical protein | *lmo1914* | -1.1 | hypothetical protein |
| *lmo2474* | 1.1 | hypothetical protein | *lmo0213* | -1.1 | peptidyl-tRNA hydrolase |
| *lmo0817* | 1.1 | PhnB protein | *lmo0708* | -1.1 | flagellar protein |
| *lmo2222* | 1.1 | hypothetical protein | *lmo0939* | -1.1 | hypothetical protein |
| *lmo0773* | 1.1 | alcohol dehydrogenase | *lmo0243* | -1.1 | RNA polymerase factor sigma-70 |
| *lmo2368* | 1.1 | hypothetical protein | *lmo1238* | -1.1 | ribonuclease PH |
| *lmo0608* | 1.1 | ABC transporter ATP-binding protein | ***lmo1701*** | -1.1 | hypothetical protein |
| *lmo1397* | 1.1 | competence damage-inducible protein CinA | *lmo2419* | -1.1 | ABC transporter ATP-binding protein |
| ***lmo0408*** | 1.1 | hypothetical protein | ***lmo1703*** | -1.1 | RNA methyltransferase |
| *lmo1956* | 1.1 | Fur family transcriptional regulator | *lmo2416* | -1.1 | hypothetical protein |
| *lmo1233* | 1.1 | thioredoxin | *lmo0706* | -1.1 | flagellar hook-associated protein FlgL |
| *lmo1975* | 1.1 | DNA polymerase IV | *lmo1450* | -1.0 | DEAD/DEAH box helicase |
| *lmo2397* | 1.1 | NifU protein | *lmo2129* | -1.0 | hypothetical protein |
| ***lmo0958*** | 1.1 | GntR family transcirptional regulator | *lmo2517* | -1.0 | hypothetical protein |
| *lmo0770* | 1.1 | LacI family transcriptional regulator | *lmo2469* | -1.0 | amino acid transporter |
| *lmo2109* | 1.1 | hydrolase | *lmo0807* | -1.0 | spermidine/putrescine ABC transporter ATP-binding protein |
| *lmo2089* | 1.1 | lipase | *lmo1582* | -1.0 | hypothetical protein |
| *lmo0928* | 1.1 | 3-methyladenine DNA glycosylase | *lmo1722* | -1.0 | ATP-dependent RNA helicase |
| *lmo1382* | 1.1 | hypothetical protein | *lmo0403* | -1.0 | hypothetical protein |
| *lmo1611* | 1.1 | aminopeptidase | *lmo0369* | -1.0 | hypothetical protein |
| *lmo0799* | 1.0 | hypothetical protein | *lmo1584* | -1.0 | hypothetical protein |
| *lmo1795* | 1.0 | hypothetical protein | *lmo0892* | -1.0 | serine phosphatase |
| *lmo1069* | 1.0 | hypothetical protein | *lmo1488* | -1.0 | nicotinic acid mononucleotide adenylyltransferase |
| *lmo2452* | 1.0 | carboxylesterase | *lmo1700* | -1.0 | hypothetical protein |
| *lmo1650* | 1.0 | hypothetical protein | *lmo1762* | -1.0 | hypothetical protein |
| *lmo0020* | 1.0 | GntR family transcriptional regulator | *lmo1845* | -1.0 | hypothetical protein |
| *lmo2461* | 1.0 | RNA polymerase factor sigma-54 | *lmo1385* | -1.0 | hypothetical protein |
| *lmo1416* | 1.0 | hypothetical protein | *lmo0453* | -1.0 | hypothetical protein |
| *lmo1683* | 1.0 | Fur family transcriptional regulator |  |  |  |
| *lmo1902* | 1.0 | 3-methyl-2-oxobutanoate hydroxymethyltransferase |  |  |  |
| ***lmo2459*** | 1.0 | glyceraldehyde-3-phosphate dehydrogenase |  |  |  |
| ***lmo1422*** | 1.0 | glycine/betaine ABC transporter permease |  |  |  |
| *lmo1741* | 1.0 | histidine kinase |  |  |  |
| *lmo1038* | 1.0 | hypothetical protein |  |  |  |
| *lmo1406* | 1.0 | pyruvate formate-lyase |  |  |  |
| *lmo1459* | 1.0 | glycyl-tRNA synthetase subunit alpha |  |  |  |

SigB-dependent genes identified by Liu et al. (2) are in bold.

**Reference for supplemental material**

1. Guerreiro DN, Wu J, McDermott E, Garmyn D, Dockery P, Boyd A, Piveteau P, O’Byrne CP. 2022. *In vitro* evolution of *Listeria monocytogenes* reveals selective pressure for loss of SigB and AgrA function at different incubation temperatures. Appl Environ Microbiol 88:e00330-22.

2. Liu Y, Orsi RH, Gaballa A, Wiedmann M, Boor KJ, Guariglia-Oropeza V. 2019. Systematic review of the *Listeria monocytogenes* σ^B^ regulon supports a role in stress response, virulence and metabolism. Future Microbiology 14:801–828.
